# Influence of sex and gonadal hormones on pain perception in a neuropathic pain model in old rats

**DOI:** 10.1590/1414-431X2026e15116

**Published:** 2026-08-07

**Authors:** C.C.A. Palmeira, I.D.G. Gaudêncio, L.O. Contiero, J.O. Barbosa, D.C. Munhoz, H.A. Ashmawi

**Affiliations:** 1 Equipe de Controle de Dor, Divisão de Anestesia, Hospital das Clínicas, Faculdade de Medicina, Universidade de São Paulo, São Paulo, SP, Brasil; 2 Faculdade de Medicina, Universidade Nove de Julho, São Paulo, SP, Brasil; 3 Centro de Ciências da Saúde, Universidade Estadual do Maranhão, São Luís, MA, Brasil; 4 Departamento de Anestesiologia, Faculdade de Ciências Médicas, Universidade Estadual de Campinas, Campinas, SP, Brasil

**Keywords:** Aging, Animal behavior, Pain behavior, Sex, Gonadal hormones, Rats, Wistar

## Abstract

Despite growing evidence that nociceptive stimuli are processed and modulated by distinct neural mechanisms depending on biological sex and aging, these factors remain underrepresented in experimental and clinical pain research. Most preclinical studies continue to rely on young male rodents, overlooking potential differences in neuropathic pain perception between sexes and age groups. Understanding these variations is critical for advancing pain management strategies. Aged rats (22 months old) of both sexes were divided into three groups: orchiectomized (Orch) males, non-orchiectomized (Non-orch) males, and females. The animals underwent L5 spinal nerve ligation and were assessed for mechanical allodynia on the plantar surface of their left hind paw before nerve ligation and on days 7, 14, 21, and 28 post-ligation. By day 14, Non-orch males showed a significantly higher withdrawal threshold than Orch males, with an average difference of 8.46 g (P<0.005), which remained significant and increased over time, peaking at day 28, with 13.07 g (P<0.0006). By day 28, Non-orch males also showed greater mechanical allodynia than females, with an estimated mean difference of approximately 8 g (P=0.038). These findings suggest that male gonadal hormones mitigate neuropathic pain-induced mechanical allodynia. The study underscores the role of testosterone in pain modulation and highlights the need for further research on the interplay between hormonal regulation, aging, and neuropathic pain mechanisms.

## Introduction

Differences between men and women in pain prevalence, pain-related behaviors, responses to treatments, and pain perception throughout the aging process have long been recognized. A substantial body of data has been collected on sex differences in pain responses, pain thresholds, pain tolerance, and responses to pain treatments (1-3). However, little is known about the effects of aging on pain, particularly regarding the influence of sex differences in older individuals.

Scientific literature on sex differences in pain perception shows that women tend to report more intense, frequent, and persistent pain than men (4-6). Data from epidemiological studies also confirm the prevalence and characteristics of chronic pain among sexes and genders (5,6). These differences between women and men are influenced by genetic, social, psychological, and hormonal factors. Hormonal factors, like gonadal hormones, are believed to be among the mechanisms underlying sex differences in pain perception (5,7-10). Gonadal hormones are related to different pain perception in animals (11), and testosterone seems to have an analgesic role (3,12). In aged subjects and those in menopause, when female gonadal hormone secretion decreases, questions arise about differences in pain perception between sexes (13). Aging seems to change pain thresholds (14,15), with men presenting higher pain thresholds for mechanical stimuli; however, studies on neuropathic pain models are scarce.

Discussions over the existence of sex differences regarding pain are ongoing, and debates on the association between pain and aging are emerging. Experimental models addressing the effects of aging on pain perception remain largely unexplored, particularly in neuropathic pain. It is well established that pain perception changes with age. Neuropathic pain seems to increase with age (16-18), and responses to acute pain stimuli are prolonged in older rats (19). While findings suggest that neuropathic pain may worsen with aging, most studies have been conducted in male rats, excluding females and thus underscoring the need to investigate potential differences in pain perception between old and young female rats.

To date, the role of gonadal hormones in neuropathic pain in aged rats has not been addressed. This study aimed to evaluate their effect on mechanical allodynia using a spinal nerve ligation model in aged rats of both sexes.

## Material and Methods

### Animals

Experiments were conducted following the approval of the Ethics Committee for Research Project Analysis at the Hospital das Clínicas da Faculdade de Medicina da Universidade de São Paulo, under number 130/10, in accordance with the International Association for the Study of Pain (IASP) guidelines on ethical research (20). All experiments were performed on male and female Wistar rats supplied by the breeding facility of the Faculdade de Medicina da Universidade de São Paulo (Brazil). Aged male and female rats (22 months old) were included. Behavioral experiments were conducted between 9:00 a.m. and 3:00 p.m. All animals were housed in cages with bedding and had free access to food and water. Only healthy aged animals were included in the study. Each cage housed two rats. Behavioral tests described here were conducted by the same rater, who was blinded to group assignments to minimize inter-rater variability.

### Experimental groups

A total of 33 animals were used, divided into three groups: old orchiectomized males (Orch), old non-orchiectomized males (Non-orch), and old females. Each group contained 7 to 11 animals. All surgeries, spinal nerve ligations, and orchiectomies were performed by the same researcher (CCAP), and all behavioral studies were performed by the same person (HAA), who was blinded to group assignment.

### Experimental procedures

#### Anesthetic technique

All surgical procedures were performed under general anesthesia with isoflurane in an induction chamber and maintained with a nasal mask at concentrations of 2-4% in 100% oxygen.

#### Bilateral orchiectomy

Male rats, under general anesthesia, were placed in supine position. After local disinfection with povidone-iodine, a 1-cm midline incision was made in the scrotum. The testicles were accessed through the opened vaginal tunic. After identifying the testicle, it was removed along with the ipsilateral *ductus deferens*. The procedure was performed on both testicles, and the tunic and skin were sutured with 4-0 nylon thread. At the end of anesthesia, animals received 0.1 mg of subcutaneous morphine for analgesia. Skin sutures were removed under general anesthesia five days post-orchiectomy, and behavioral experiments were conducted 14 days after orchiectomy, when testosterone levels were very low (21).

### L5 spinal nerve ligation

Under general anesthesia, the rats were placed in the prone position, and the lumbar region was shaved. A skin incision was made, the paravertebral muscles were separated, and the spinous processes of L4, L5, and L6 were identified. The L5 and L6 spinous processes were excised to expose the dorsal roots. The left L5 spinal nerve was ligated using 6-0 silk and cut 1 mm distal to the ligature. After achieving hemostasis, the incision was sutured with 4-0 nylon. Before recovery from anesthesia, the animals received 0.1 mg of subcutaneous morphine for postoperative analgesia. Under general anesthesia, skin sutures were removed on the fifth day after surgery. Animals exhibiting altered gait were excluded from the study (n=6). Paw withdrawal thresholds following mechanical stimuli were assessed on the left hind paw (22).

### Mechanical allodynia

Paw withdrawal threshold was measured using electronic von Frey anesthesiometer (Insight Ltda., Brazil). Rats were placed in elevated, transparent acrylic cages (21×27×15 cm) with nylon mesh flooring (12×12 mm) that allowed free access to the plantar surfaces of the hind paws. The stimulus was applied five times at 4-min intervals from beneath the mesh floor, perpendicular to the plantar surface of the left hind paw, and the mean value was calculated. The response was defined as withdrawal of the stimulated paw. A cutoff value of 60 g was used to avoid tissue damage.

Before all behavioral evaluations, animals were acclimated to the observation chamber environment for 20 min. Paw withdrawal thresholds to mechanical stimuli were assessed before ligation and on postoperative days 7, 14, 21, and 28 (POD7, POD14, POD21, and POD28) after nerve ligation.

### Sample size calculation

The sample size was calculated to detect differences of 8 units (g) from female rats, with 80% power and a type 1 error of 5%, considering a standard deviation of 6 units (g), obtained in our previous results using adult rats, with a minimum of 7 animals per group.

### Statistical analysis

Continuous variables were analyzed using means, medians, standard deviations, and quartiles. A mixed model with random effects for repeated measurements over time and an interaction parameter between time and group was used to evaluate group effects on the measures. P-values <0.05 were regarded as statistically significant. Data analysis was performed using the R 4.0.2 program (R Care Team, 2020).

## Results

**Figure 1:**
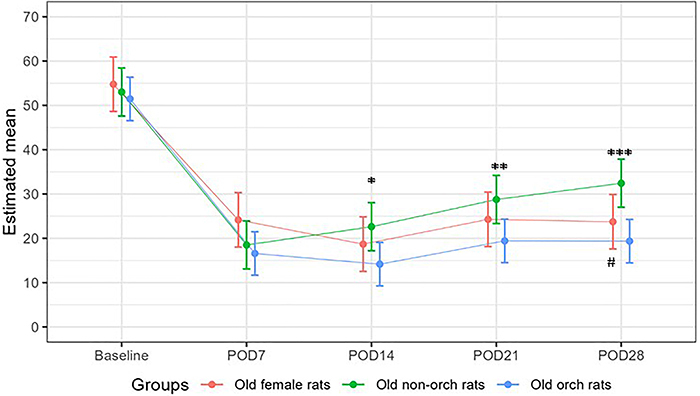
Results of the mechanical allodynia test in old female rats, orchiectomized (Orch) male rats, and non-orchiectomized (Non-orch) male rats. Data are reported as mean and SD. *P=0.0239, **P=0.0128, ***P=0.0006 between Orch and Non-orch groups; ^#^P=0.038 between Orch and female groups (ANOVA).

Mechanical allodynia was observed in all groups, appearing on POD7 and remaining at similar levels until POD28. The effect was present regardless of sex or the presence of gonads (Table 1 and Figure 1).

**Table 1:** Paw withdrawal threshold (g) in aged female rats, orchiectomized male rats, and non-orchiectomized male rats over time.

Time points	Aged orchiectomized rats	Aged non-orchiectomized rats	Aged female rats
Baseline			
Mean±SD	51.5±4.9 (n=11)	53.0±3.9 (n=9)	54.8±4.3 (n=7)
Median [quartile]	52.7 [50.5; 54.5]	51.3 [51.3; 54.4]	56.9 [53.5; 57.3]
POD7			
Mean±SD	16.6±7.9 (n=11)	18.5±7.2 (n=9)	24.2±5.6 (n=7)
Median [quartile]	14.7 [8.9; 21.0]	17.6 [12.8; 21.9]	22.3 [21.7; 27.5]
POD14			
Mean±SD	14.2±7.6 (n=11)	22.6±8.9 (n=9)	18.7±6.4 (n=7)
Median [quartile]	12.2 [9.0; 17.5]	26.2 [14.8; 30.4]	19.4 [14.4; 21.6]
POD21			
Mean±SD	19.4±10.0 (n=11)	28.8±15.5 (n=9)	24.3±9.3 (n=7)
Median [quartile]	15.8 [11.8; 28.2]	26.9 [18.4; 30.4]	21.9 [19.5; 25.1]
POD28			
Mean±SD	19.4±8.1 (n=11)	32.4±8.3 (n=9)	23.7±6.7 (n=7)
Median [quartile]	15.5 [13.1; 26.6]	33.1 [29.5; 37.1]	22.9 [20.0; 27.6]

POD: post-operative day.

Differences among groups appeared on POD14. Withdrawal threshold after mechanical stimulus was lower in the Orch group than in the Non-orch group, with an estimated difference of 8.46 g (P=0.0239). The difference in withdrawal threshold increased over time and reached 13.07 g lower than in Non-orch animals by POD28 (P=0.0006), when the mean withdrawal threshold in orchiectomized rats was around 67% higher than in non-orchiectomized rats (19.4±8.1 and 32.4±8.3 g). On day 28, Non-orch rats also showed a higher withdrawal threshold than female rats, with an estimated mean difference of approximately 8 g between the groups. No significant difference was observed between female and orchiectomized rats during the entire observation period. Table 2 describes pairwise multiple comparisons between groups at each time point, along with the estimated mean differences.

**Table 2:** Pairwise multiple comparisons of aged female rats, orchiectomized (Orch) male rats, and non-orchiectomized (Non-orch) male rats at each time point.

Comparison	Estimated difference	95%CI		P
		Lower	Upper	
Baseline				
Female × Non-orch rats	1.75	-6.5	9.9	0.6727
Female × Orch rats	3.31	-4.6	11.2	0.4058
Non-orch × Orch rats	1.56	-5.8	8.9	0.6730
POD7				
Female × Non-orch rats	5.66	-2.5	13.9	0.1739
Female × Orch rats	7.58	-0.3	15.4	0.0589
Non-orch × Orch rats	1.92	-5.4	9.2	0.6036
POD14				
Female × Non-orch rats	-3.94	-12.1	4.3	0.3428
Female × Orch rats	4.52	-3.3	12.4	0.2567
Non-orch × Orch rats	8.46	1.1	15.8	0.0239
POD21				
Female × Non-orch rats	-4.49	-12.7	3.7	0.2800
Female × Orch rats	4.86	-3	12.7	0.2231
Non-orch × Orch rats	9.34	2	16.7	0.0128
POD28				
Female × Non-orch rats	-8.69	-16.9	-0.5	0.038
Female × Orch rats	4.38	-3.5	12.2	0.2717
Non-orch × Orch rats	13.07	5.8	20.4	0.0006

POD: post-operative day. Mixed model with random effects for repeated measurements.

## Discussion

This study investigated the role of sex and gonadal hormones in pain behavior elicited by mechanical stimuli in aged rats following spinal nerve ligation. Uncertainties remain regarding the components modulating pain responses between sexes, especially in older animals, which are less studied. Male rats had either intact gonads or were orchiectomized, and female rats were in reproductive senescence. Our findings showed that male gonads play an important role in increasing the pain threshold following mechanical stimulation in the neuropathic pain model used.

Most research on experimental pain models has primarily been conducted in young male rats, including studies on neuropathic pain (23-25). This preference for young males is attributed to hormonal stability, as females experience significant hormonal fluctuations due to the estrous cycle (26), but the need to study females has surpassed this old concept.

Although studies with both sexes are increasing, the effect of age has not been properly studied and research on aged females remains limited (16-19). We found that gonadal hormones exert an antinociceptive role in older male animals, as older orchiectomized rats showed significant decreases in paw withdrawal thresholds, suggesting that the presence of testosterone plays a crucial role in antinociception. Our findings are in line with previous studies (3,27), showing a difference in pain threshold of around 67% due to the presence of male gonad, where non-orchiectomized rats presented a much higher withdrawal threshold than orchiectomized rats after four weeks of spinal ligation. Several mechanisms for the antinociceptive effect of testosterone have been demonstrated, such as inhibition of the serotonin transporter (SERT) in the synaptic cleft (28), decrease of cold allodynia mediated by TRPM8 (29), decreased production of pro-inflammatory cytokines like TNF-α induced by testosterone (30), and decreased capsaicin receptor-mediated signaling in dorsal root ganglion neurons (31). Interestingly, the antinociceptive effect remained in aged animals, since orchiectomized rats presented more pain behavior than intact male rats.

Research in neuropathic rats suggests that thermal and mechanical pain sensitivity increases with age, which can lead to greater susceptibility to depression and cognitive decline associated with chronic pain in middle-aged animals (16-18). We did not compare mechanical pain sensitivity across different ages, focusing only on the aged population, in which intact male animals presented less pain behavior.

This study is the first to address the effects of sex, gonadal hormones, and aging in rats using a behavioral model of neuropathic pain. The less intense mechanical allodynia observed in older, non-orchiectomized male rats suggests that testosterone plays a significant protective role in neuropathic pain modulation. These findings provide new insights into the role of gonadal hormones in pain perception, especially in aging animals.

One limitation of this study was not obtaining serum male and female gonadal hormone levels. It is known that adult rats after orchiectomy present low serum testosterone levels (21), and our findings indicated that the absence of testosterone leads to decreased pain tolerance in elderly male rats. This suggests that the testosterone levels present in elderly non-castrated animals are sufficient to modulate pain. This challenges the traditional view that sex alone is the primary determinant of pain threshold. These results have important implications for understanding hormonal mechanisms in pain modulation and for developing new therapeutic strategies for managing neuropathic pain in older populations. Another limitation is that we assumed the rats were in reproductive senescence, which begins around 13 months of age (32).

The clinical relevance of these findings may be significant. In humans, testosterone replacement therapy is used to treat symptoms of testosterone deficiency in older men. It has well-known effects on sexual function, muscle mass, and bone density (33). Now there is a new perspective for research on the role of testosterone for treating neuropathic pain in older men.

Our findings suggest that male gonadal hormones mitigate neuropathic pain-induced mechanical allodynia. The study underscores the role of testosterone in pain modulation and highlights the need for further research on the interplay among hormonal regulation, aging, and neuropathic pain mechanisms.

## Data Availability

All data generated or analyzed during this study are included in this published article.

## References

[1] Racine M, Tousignant-Laflamme Y, Kloda LA, Dion D, Dupuis G, Choiniàre M (2012). A systematic literature review of 10 years of research on sex/gender and pain perception - part 1: are there really differences between women and men?. Pain.

[B02] Racine M, Tousignant-Laflamme Y, Kloda LA, Dion D, Dupuis G, Choiniàre M (2012). A systematic literature review of 10 years of research on sex/gender and pain perception - part 2: do biopsychosocial factors alter pain sensitivity differently in women and men?. Pain.

[3] Barbosa JO, Garcia JBS, Cartágenes MSS, Amaral AG, Onuchic LF, Ashmawi HA (2019). Influence of androgenic blockade with flutamide on pain behaviour and expression of the genes that encode the NaV1.7 and NaV1.8 voltage-dependent sodium channels in a rat model of postoperative pain. J Transl Med.

[4] Fillingim RB, King CD, Ribeiro-Dasilva MC, Rahim-Williams B, Riley JL (2009). Sex, gender, and pain: a review of recent clinical and experimental findings. J Pain.

[B05] Dahlhamer J, Lucas J, Zelaya C, Nahin R, Mackey S, DeBar L (2018). Prevalence of chronic pain and high-impact chronic pain among adults - United States, 2016. MMWR Morb Mortal Wkly Rep.

[6] Mogil JS (2012). Sex differences in pain and pain inhibition: multiple explanations of a controversial phenomenon. Nat Rev Neurosci.

[7] Aloisi AM, Ceccarelli I, Herdegen T (2000). Gonadectomy and persistent pain differently affect hippocampal c-Fos expression in male and female rats. Neurosci Lett.

[B08] Greenspan JD, Craft RM, LeResche L, Arendt-Nielsen L, Berkley KJ, Fillingim RB (2007). Studying sex and gender differences in pain and analgesia: a consensus report. Pain.

[B09] Hurley RW, Adams MCB (2008). Sex, gender and pain: an overview of a complex field. Anesth Analg.

[10] Thompson AD, Angelotti T, Nag S, Mokha SS (2008). Sex-specific modulation of spinal nociception by alpha-adrenoceptors: differential regulation by estrogen and testosterone. Neuroscience.

[11] Palmeira CCA, Ashmawi HA, Posso IP (2011). Sex and pain perception and analgesia. Rev Bras Anestesiol.

[12] Aloisi AM, Ceccarelli I, Fiorenzani P, De Padova AM, Massafra C (2004). Testosterone affects formalin-induced responses differently in male and female rats. Neurosci Lett.

[13] Gibson CJ, Li Y, Bertenthal D, Huang AJ, Seal KH (2019). Menopause symptoms and chronic pain in a national sample of midlife women veterans. Menopause.

[14] Petrini L, Matthiesen ST, Arendt-Nielsen L (2015). The effect of age and gender on pressure pain thresholds and suprathreshold stimuli. Perception.

[15] Girotti G, Trevisan C, Fratta S, Toffanello ED, Inelmen EM, Manzato E (2019). The impact of aging on pressure pain thresholds: are men less sensitive than women also in older age?. Eur Geriatr Med.

[16] Leite-Almeida H, Almeida-Torres L, Mesquita AR, Pertovaara A, Sousa N, Cerqueira JJ (2009). The impact of age on emotional and cognitive behaviours triggered by experimental neuropathy in rats. Pain.

[B17] Kim YI, Na HS, Yoon YW, Nahm SH, Ko KH, Hong SK (1995). Mechanical allodynia is more strongly manifested in older rats in an experimental model of peripheral neuropathy. Neurosci Lett.

[B18] Crisp T, Giles JR, Cruce WL, McBurney DL, Stuesse SL (2003). The effects of aging on thermal hyperalgesia and tactile-evoked allodynia using two models of peripheral mononeuropathy in the rat. Neurosci Lett.

[19] ickering G, Jourdan D, Millecamps M, Chapuy E, Alliot J, Eschalier A (2006). Age-related impact of neuropathic pain on animal behaviour. Eur J Pain.

[20] Zimmermann M (1983). Ethical guidelines for investigations of experimental pain in conscious animals. Pain.

[21] Kyprianou N, Isaacs JT (1987). Biological significance of measurable androgen levels in the rat ventral prostate following castration. Prostate.

[22] Kim SH, Chung JM (1992). An experimental model for peripheral neuropathy produced by segmental spinal nerve ligation in the rat. Pain.

[23] Robinson I, Meert TF (2005). Stability of neuropathic pain symptoms in partial sciatic nerve ligation in rats is affected by suture material. Neurosci Lett.

[B24] Ossipov MH, Bazov I, Gardell LR, Kowal J, Yakovleva T, Usynin I (2007). Control of chronic pain by the ubiquitin proteasome system in the spinal cord. J Neurosci.

[25] Obara I, Parkitna JR, Korostynski M, Makuch W, Kaminska D, Przewlocka B (2009). Local peripheral opioid effects and expression of opioid genes in the spinal cord and dorsal root ganglia in neuropathic and inflammatory pain. Pain.

[26] Dina OA, Gear RW, Messing RO, Levine JD (2007). Severity of alcohol-induced painful peripheral neuropathy in female rats: role of estrogen and protein kinase (A and Cε). Neuroscience.

[27] Borzan J, Fuchs PN (2006). Organizational and activational effects of testosterone on carrageenan-induced inflammatory pain and morphine analgesia. Neuroscience.

[28] Plumb AN, Lesnak JB, Rasmussen L, Sluka KA (2025). Female specific interactions of serotonin and testosterone in the rostral ventromedial medulla after activity-induced muscle pain. J Pain.

[29] Khalilzadeh E, Aliyoldashi M, Abdkarimi B, Azarpey F, Vafaei Saiah G, Hazrati R (2023). Reversal of cold intolerance by testosterone in orchiectomized mice after tibial nerve transection. Behav Brain Res.

[30] Lenert ME, Avona A, Garner KM, Barron LR, Burton MD (2021). Sensory neurons, neuroimmunity, and pain modulation by sex hormones. Endocrinology.

[31] Gupta S, McCarson KE, Welch KM, Berman NE (2011). Mechanisms of pain modulation by sex hormones in migraine. Headache.

[32] Lu KH, Hopper BR, Vargo TM, Yen SS (1979). Chronological changes in sex steroid, gonadotropin and prolactin secretions in aging female rats displaying different reproductive states. Biol Reprod.

[33] Basaria S, Coviello AD, Travison TG, Storer TW, Farwell WR, Jette AM (2010). Adverse events associated with testosterone administration. N Engl J Med.

